# Molecular Basis of Ligand Dissociation in β-Adrenergic Receptors

**DOI:** 10.1371/journal.pone.0023815

**Published:** 2011-09-07

**Authors:** Angel González, Tomas Perez-Acle, Leonardo Pardo, Xavier Deupi

**Affiliations:** 1 Laboratori de Medicina Computacional, Unitat de Bioestadística, Facultat de Medicina, Universitat Autònoma de Barcelona, Bellaterra, Catalunya, Spain; 2 Departamento de Ciencias Biológicas, Facultad de Ciencias Biológicas, Universidad Andrés Bello, Santiago, Chile; 3 Computational Biology Lab, Center for Mathematical Modeling, Facultad de Ciencias Físicas y Matemáticas, Universidad de Chile, Santiago, Chile; 4 Centro Interdisciplinario de Neurociencias de Valparaíso, Playa Ancha, Valparaíso, Chile; 5 Fundación Ciencia para la Vida, Ñuñoa, Santiago, Chile; 6 Condensed Matter Theory Group and Laboratory of Biomolecular Research, Paul Scherrer Institut, Villigen PSI, Switzerland; University of Cambridge, United Kingdom

## Abstract

The important and diverse biological functions of β-adrenergic receptors (βARs) have promoted the search for compounds to stimulate or inhibit their activity. In this regard, unraveling the molecular basis of ligand binding/unbinding events is essential to understand the pharmacological properties of these G protein-coupled receptors. In this study, we use the steered molecular dynamics simulation method to describe, in atomic detail, the unbinding process of two inverse agonists, which have been recently co-crystallized with β_1_ and β_2_ARs subtypes, along four different channels. Our results indicate that this type of compounds likely accesses the orthosteric binding site of βARs from the extracellular water environment. Importantly, reconstruction of forces and energies from the simulations of the dissociation process suggests, for the first time, the presence of secondary binding sites located in the extracellular loops 2 and 3 and transmembrane helix 7, where ligands are transiently retained by electrostatic and Van der Waals interactions. Comparison of the residues that form these new transient allosteric binding sites in both βARs subtypes reveals the importance of non-conserved electrostatic interactions as well as conserved aromatic contacts in the early steps of the binding process.

## Introduction

G protein-coupled receptors (GPCRs) represent one of the largest protein families in mammals [1] and constitute 2%–3% of the human proteome [2]. GPCRs transduce sensory signals of external origin, such as photons, odors or pheromones, and endogenous signals including biogenic amines, (neuro)peptides, proteases, glycoprotein hormones and ions, into the cell. Thus, these receptors are essential in cell physiology, and their malfunction is commonly translated into pathological outcomes [3]. As a result, GPCRs constitute one of the most important pharmaceutical targets, as around 40% of prescribed drugs act through this family of proteins [4]. These receptors feature a common fold of seven transmembrane helices (TMs 1 to 7) connected by three extracellular (ECLs 1 to 3) and three intracellular (ICLs 1 to 3) loops [5], with an extracellular N-terminus and an intracellular C-terminus. Extracellular regions are very diverse in structure and amino acid composition, and in many GPCRs, as glycoprotein hormone and peptide receptors in family A or most receptors in families B and C, they are directly involved in ligand binding [6]. While smaller ligands bind in a pocket relatively buried within the TM bundle, they must also interact with the extracellular regions in order to reach the binding site. Understanding the molecular basis of ligand-receptor interactions in the extracellular domains is of great importance, as they are implicated in many aspects of receptor function, as ligand binding [7] and specificity [8], allosterism [9] or receptor activation [10], [11]. Importantly, recent NMR data show ligand-specific conformational changes in the extracellular surface of the β_2_-adrenergic receptor (β_2_AR) [12].

While there is a vast amount of pharmacological, functional and pathophysiological information about GPCRs deposited in specialized databases (e.g. IUPHAR-DB, at http://www.iuphar-db.org), structural data of GPCRs is still scarce. Presently, only the structures of eight Class A GPCRs (bovine and squid rhodopsins, human β_2_-adrenergic, turkey β_1_-adrenergic, human A_2A_ adenosine (reviewed in [13], [14], [15]), human CXCR4 chemokine [16], human dopamine D_3_ [17] and human histamine H_1_ [18] receptors) are known. The availability of the structure of the β_1_AR [19] and β_2_AR [20] represents a unique opportunity to investigate the similarities and/or differences in the ligand entry process between these closely related subtypes. While these receptors have slightly different pharmacological properties [21], they present a strong similarity in sequence and structure, particularly in the TM bundle and orthosteric binding pockets [19]. Thus, it is plausible to argue that extracellular regions can have an impact on the different pharmacological properties between subtypes. Previous theoretical studies, using random acceleration molecular dynamics simulations, have suggested that ligands access the orthosteric binding site of the β_2_AR mainly through an opening at the extracellular surface [22]. Conversely, ligand docking calculations in opsin located the paths for access/egress between transmembrane helices [23]. This difference is due to both the different architecture of the extracellular regions and the different chemical nature of their respective ligands. While the β_2_AR binding pocket is relatively exposed to the solvent, ECL 2 and the N-terminal of opsin cover the binding pocket, which form a “plug” that prevents the access of the ligand from the extracellular environment.

In this work, we have conducted a comparative analysis of the process of ligand dissociation in β_1_ and β_2_ARs using the steered molecular dynamics (SMD) simulation method [24]. SMD has been very successful in the study of dissociation reactions of several small-molecules/protein complexes through application of external forces on nanosecond time scales [25], [26], [27], [28], and is particularly useful to describe the interactions occurring in the binding/unbinding of ligands [25]. Our results suggest that both receptors have two putative ligand entry channels located at the extracellular region, discarding the entry channels located between the transmembrane segments that lead to the lipid environment. By monitoring the forces and energies of the ligand-dissociation along these extracellular channels in both βAR structures, we have identified for the first time two secondary binding pockets in the extracellular region of the receptors. In addition, we discuss the importance for the ligand exit/entry process of non-conserved charged residues and conserved aromatic interactions shared by the two entry channels.

## Results

### Ligand entry/exit channels in β_1_ and β_2_ adrenergic receptors

**Figure 1:**
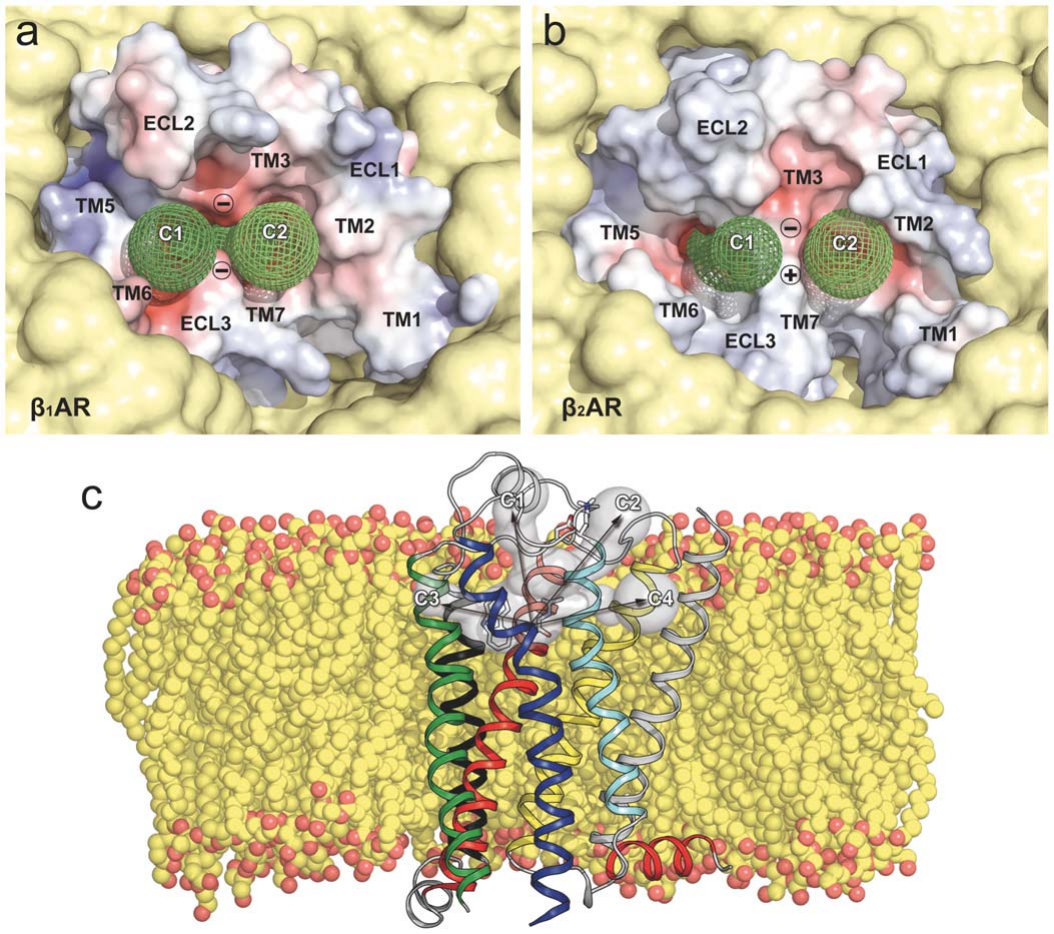
Extracellular molecular surfaces of the β_1_AR (panel a) and β_2_AR (panel b), embedded in a lipid bilayer (in yellow). The electrostatic potential was calculated using the program APBS with nonlinear Poisson-Boltzmann equation and contoured at ±10 kT/e (negatively and positively charged surface areas in red and blue, respectively). The accessible channels (C1 and C2) identified by CAVER are depicted as green wires. D217/D356 in β_1_AR (panel a) and the salt bridge D192/K305 in β_2_AR (panel b) are represented by circled − and + symbols. Panel c displays the extraction vectors along the four channels (C1 to C4) at the end of the equilibration run. The β_2_AR ribbon structure is colored as follows: TM1 (grey), TM2 (yellow), TM3 (red), TM4 (black), TM5 (green), TM6 (blue), TM7 (cyan), and helix 8 (red). Carazolol is shown in white sticks. Pictures were prepared using PyMOL (http://www.pymol.org/).

Using the skeleton search algorithms implemented in the CAVER program [29], we explored routes that connect the buried orthosteric binding pocket to the extracellular surface in the structures of the human β_1_AR and β_2_AR. Figure 1 displays two entry channels identified in each receptor, located between TMs 3, 5, 6 and 7 (C1) and TMs 1, 2, 3 and 7 (C2). These channels are separated from each other by charged residues in ECLs 2 and 3; D217 and D356 in β_1_AR (Figure 1a) and D192 and K305, forming a salt bridge, in β_2_AR (Figure 1b). These residues, in combination with other neighboring polar/charged amino acids, confer a negative electrostatic potential to both channels, which suggests the existence of an electronegative funnel to attract positively charged ligands into the orthosteric binding site of beta adrenoceptors [30]. On the other hand, the entrance/exit channels for retinal in rhodopsin have been proposed to occur through the lipid bilayer, via two openings located between TMs 1 and 7, and TMs 5 and 6, respectively [23]. While CAVER does not detect these alternative channels in the βAR structures, in order to further assess their possible relevance, we identified these two channels on the structure of the ligand-free apoprotein opsin (PDB entry 3CAP [31]) and mapped them onto the βAR structures by coordinate superimposition (C3 and C4 in Figure 1c).

### Channel route preferences for ligand dissociation

**Figure 2:**
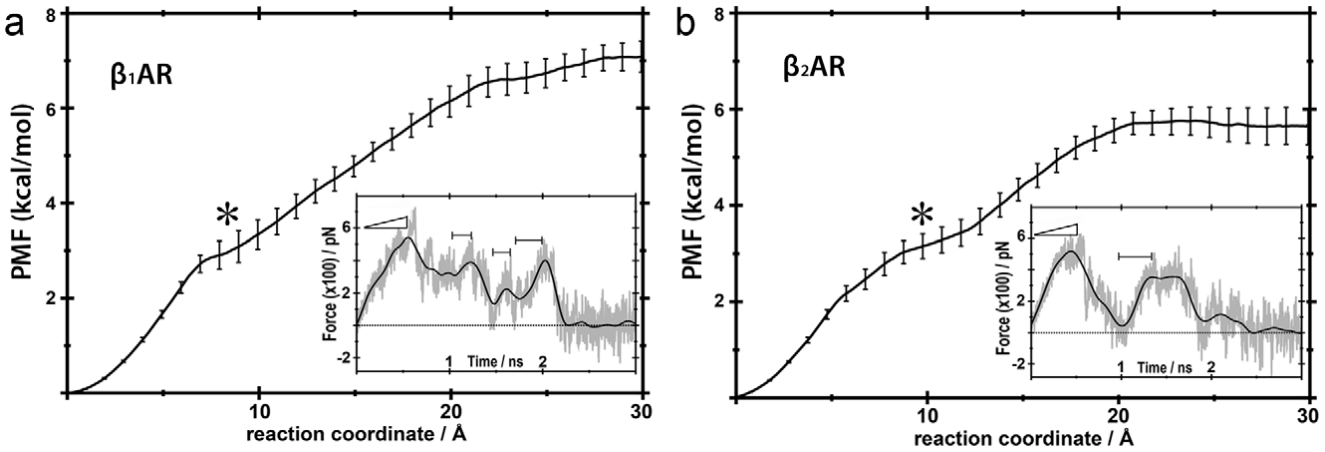
PMF and force profiles of ligand extraction along the extracellular channel C1. Panel a, dissociation of cyanopindolol from β_1_AR. Panel b, dissociation of carazolol from β_2_AR. The star symbols correspond to the snapshots depicted in Figure 5. The statistical error in the PMF data is shown in bars. Inset figures display representative force profiles of the repeated trajectories. The force simulation data is shown in grey and smoothed to a black line. Horizontal bars denote regions with positive slope in the force profile.

To study the process of ligand release from β_1_AR and β_2_AR orthosteric binding pockets, we performed SMD simulations of the antagonist-receptor complexes embedded in a model lipid bilayer (see Methods). Ten nanoseconds of equilibration were performed to obtain constant values of energy, cell volume and lipid density. The root mean square deviation (rmsd) of the protein backbone atoms from the initial coordinates during equilibration stabilizes rapidly to a value in the vicinity of 2.0 Å (Figure S1). Following this equilibration period, steered forces were applied to both ligands along the four calculated channels (C1 to C4 in Figure 1c). Figure S2 displays representative force profiles of the pulling experiments of cyanopindolol (Figure 2a) and carazolol (Figure 2b) along extracellular C1 (black) and C2 (blue) and lipid C3 (red) and C4 (yellow) channels. The initial force peaks to remove ligands from the orthosteric binding site via extracellular C1 or C2 channels were on average ∼600 pN, a typical value in ligand diffusion SMD experiments [26], [32]. On the contrary, pulling the ligands through the proposed rhodopsin channels (C3 and C4 in Figure 1c), required forces two-fold larger than for the extracellular routes. These results strongly suggest that, in βARs, the transit of molecules through the lipidic phase, via TMs 1 and 7 or TMs 5 and 6, is not favored compared to the extracellular routes. Consequently, the C3 and C4 channels were not included in the rest of the analysis.

### Residues implicated in ligand-receptor interactions during dissociation

**Figure 3:**
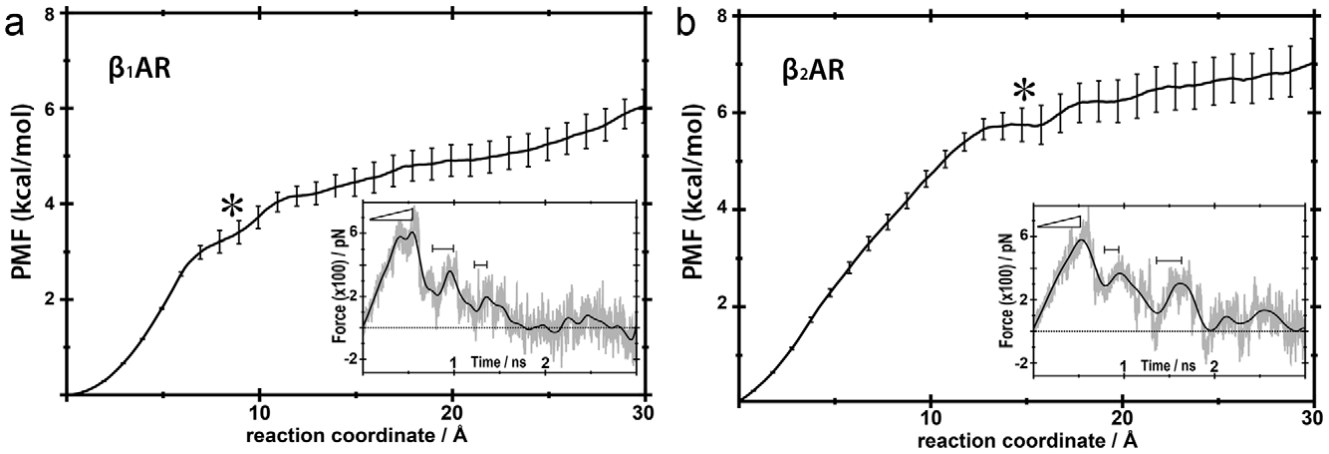
PMF and force profiles of ligand extraction along the extracellular channel C2. Panel a, dissociation of cyanopindolol from β_1_AR. Panel b, dissociation of carazolol from β_2_AR. The star symbols correspond to snapshots depicted in Figures 6. See legend of Figure 2 for details.

Figures 2 and 3 display the potential of mean force (PMF) and representative force profiles (insets) of the pulling experiments of cyanopindolol (Figures 2a and 3a) and carazolol (Figures 2b and 3b) along the extracellular C1 and C2 channels. In all cases, small fluctuations were observed in receptor structures during ligand extraction, which were in similar ranges to the rmsd values of the equilibration runs (data not shown). These results indicate that selected velocities, force constants, and extraction vectors were adequate to achieve smooth ligand releases. Thus, no steric clashes occur between molecules and receptors during dissociation. Horizontal bars in the insets of Figures 2 and 3 represent time periods of relatively strong ligand-receptor interaction during dissociation. Positive slopes in force profiles characterized these periods. Clearly, disruption of the initial interactions between the ligands and orthosteric binding site residues, which mainly include the electrostatic interaction with D^3.32^ and hydrogen bonds with N^7.39^ and S^5.42^ (superscript numbers correspond to the Ballesteros & Weinstein general numbering scheme [33]), requires a maximal force (ramp symbols in the force insets). After this primary unbinding event (∼0.5 ns), forces fall as the ligands displace further from the orthosteric binding site towards the solvent through the exit channels. Then, subsequent regions of increasing forces indicate secondary interaction sites along the channels.

**Figure 4:**
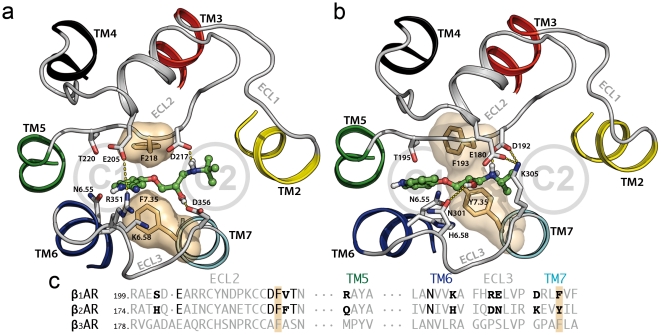
Secondary binding pockets identified in the C1 channel. Panel a shows the cyanopindolol/β_1_AR complex and panel b shows the carazolol/β_2_AR complex. The orientation of these views are the same as in Figures 1a and 1b. Circles show the approximate locations of channels C1 and C2. Ligands are shown in green sticks, and side chains within 3 Å of the ligands are shown in white sticks. Solvent-accessible surfaces of aromatic F359/Y308^7.35^ and F218/F193 residues are displayed in orange. Panel c depicts the sequence alignment of this region between human βARs. Residues along the extraction trajectories that interact with ligands are highlighted in black, and non-conserved residues are showed in a smaller size.

**Figure 5:**
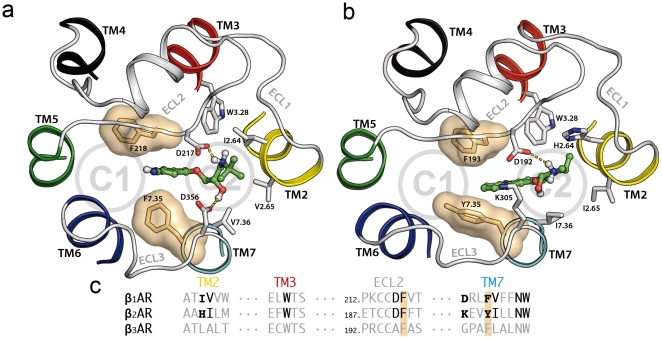
Secondary binding pockets identified in the C2 channel. Panel a shows the cyanopindolol/β_1_AR complex and panel b shows the carazolol/β_2_AR complex. See legend of Figure 4 for details.

The extraction of cyanopindolol through channel C1 in β_1_AR reveals two major retention events, (Figure 2a). In an initial step at ∼1.2 ns, cyanopindolol is stabilized by an ionic interaction between the protonated amine of the ligand and D217 in ECL2 and a hydrogen bonding interaction between the β-OH group and D356^7.32^ (Figure 4a). Later, in the final steps of its movement toward the extracellular solvent (∼2.0 ns), increasing forces are required to break a salt bridge between E205 in ECL2 and R351 in ECL3 (also shown in Figure 4a), in order to allow the ligand escape. Conversely, the extraction of carazolol from β_2_AR through C1 is characterized by a single retention site at ∼1.2 ns (Figure 2b). At this point, the protonated amine of the ligand interacts with D192 in ECL2 and the β-OH group with N301 in ECL3 (Figure 4b). Table 1 lists residues in the vicinity of the ligands during the dissociation process that form this extraction channel. On the other hand, ligands extraction through channel C2 shows two retention sites at ∼0.9 and ∼1.5 ns in both adrenoceptors (Figure 3). Initially, the protonated amine of cyanopindolol or carazolol interacts with either D217 or D192 in ECL2 of β_1_- and β_2_- receptors, respectively (Figures 5a and 5b). The second barrier corresponds to Van der Waals attractive forces between the aromatic moieties of the ligands and bulky residues at positions 2.64, 2.65, 3.28, 7.36, 7.39 and 7.40 in TMs 2, 3 and 7 (summarized in Table 1). In the final steps of the simulations the ligands drifted apart from the receptors with no further retention and the forces decays to zero.

**Table 1:** Summary of the residues that interact with the ligand during the SMD pulling experiments through C1 and C2 in β_1_ and β_2_ARs.

*C1*	*BW*	*β_1_AR*	*β_2_AR*	*β_3_AR*
*ECL2*	-	S203	H178	A182
	-	E205	E180	E185
	-	**D217**	**D192**	**A197**
	-	**F218**	**F193**	**F198**
	-	V219	F194	A199
	-	T220	T195	S200
*TM5*	5.36	R222	Q197	M202
*TM6*	6.55	N344	N293	N312
	6.58	K347	H296	R315
*ECL3*	-	R351	D300	P320
	-	E352	N301	S321
	-	**D356**	**K305**	**G325**

*C2*				
*TM2*	2.64	I118	H93	L97
	2.65	V119	I94	A98
*TM3*	3.28	W134	W109	W113
*ECL2*	-	**D217**	**D192**	**A197**
	-	**F218**	**F193**	**F198**
*TM7*	7.36	V360	I309	L329
	7.39	N363	N312	N332
	7.40	W364	W313	W333
*ECL3*	-	**D356**	**K305**	**G325**

The corresponding residues in the β_3_AR subtype are also included for comparison. The generic numbering of Ballesteros & Weinstein (BW) is shown for TM amino acids. Numbering of residues corresponds to the human annotated sequences UniProtKB/Swiss-Pro entries: β_1_AR (P08588), β_2_AR (P07550) and β_3_AR (P13945), and the residues that form part of both channels are shown in boldface.

### Physico-chemical nature and sequence conservation of the entry channels

In both β_1_- and β_2_AR, the two identified extracellular channels of ligand entry/exit differ strongly in their physico-chemical properties, as channel C1 is strongly hydrophilic (10 polar/charged residues out of 13) whereas C2 is mainly hydrophobic (7 apolar/aromatic residues out of 10) (summarized in Table 1). Despite this overall similarity between β_1_AR and β_2_AR in the fundamental nature of the ligand entry/exit routes, sequence conservation in these regions strongly differs between receptors. Comparison of conserved residues reveals that the sequence identity between β_1_- and β_2_AR in channel C1 is only 38% (5 out of 13 residues), while in C2 is 70% (7 out of 10 residues, considering Ile and Val as nearly equivalent).

### Characterization of intermediate binding sites

The potential of mean force along extraction coordinates was calculated using the second cumulant expansion of Jarzynski's expression by sampling the work from repeated trajectories [34]. PMF values between starting and ending points were used to estimate free energy changes of dissociation reactions. The free energy cost of moving the ligand from the binding site crevice to bulk water is 7.0 or 6.0 kcal/mol for β_1_AR, and 5.6 or 6.9 kcal/mol for β_2_AR, via C1 or C2 channels, respectively (Figures 2 and 3). Clearly, these positive values indicate that receptor-bound states are more favorable in both receptors. Obviously, initial (ligand bound to receptor) and final (ligand in bulk water) states of the SMD simulations, via C1 or C2 channels, are the same, allowing us to estimate the procedure error. The difference in energy of 1.0 and 1.3 kcal/mol, observed for β_1_AR and β_2_AR, respectively, between channels C1 and C2, are considered small errors given the complexity of the ligand-receptor-lipid bilayer system. Although no energy minimum was found in the free energy profile, we observed a decrease in the PMF slopes in a narrow region, at distance of ∼9 to 15 Å from the orthosteric binding sites in all experiments (black stars in Figure 2 and 3). These secondary binding pockets correlate with the retention regions identified previously in the C1 and C2 channels and comprise residues located in ECL2 and ECL3, and in the outermost solvent exposed area of TMs (Figures 4 and 5). The free energy cost to move cyanopindolol from the orthosteric binding pocket of β_1_AR to the secondary binding pocket situated in C1 (2.9 Kcal/mol) is comparable to the value found for the C2 channel (3.2 kcal/mol) and both are located at a distance of ∼9.0 Å from the orthosteric binding site. In contrast, the secondary binding pocket in C2 (5.7 kcal/mol) of β_2_AR is less favorable than in the C1 channel (3.1 kcal/mol) and is located at ∼15 Å from the orthosteric binding site. In this particular case, additional energy is required to displace the bulky carbazole group of carazolol through the bulky H^2.64^, I^2.65^, W^3.28^ and I^7.36^ residues in TMs 2, 3 and 7 (Figure 5b).

## Discussion

In this work, we have explored the possible exit routes of ligands in the structures of human β_1_AR and β_2_AR using SMD simulations. We have found that both receptors have two well-defined access channels from the extracellular side (C1 and C2 in Figure 1). While we explicitly simulate the process of ligand dissociation, the relatively rigid arrangement of the extracellular domains of the receptors strongly suggests that the same channels are also used in the process of ligand entry. During dissociation, ligands are retained in the boundary with the extracellular solvent (∼9–15 Å from the orthosteric binding site, Figures 4 and 5), as evidenced by the decrease in the PMF slopes and larger force values during the SMD experiments (Figure 2 and 3, black stars). We suggest that these retention sites serve as secondary binding pockets during ligand entry. Interestingly, the access channels differ strongly in their physicochemical properties and, particularly, in their degree of sequence conservation (38% identity in C1 vs. 70% identity in C2). However, our simulations produce similar PMF profiles for C1 and C2 in both receptors and, thus, both routes may serve indistinguishably for the entry and exit of inverse agonists. Importantly, all the TM residues identified in our study have been experimentally found to be involved in ligand interactions for βARs or/and other GPCRs: 2.64 [35], [36], 2.65 [37], [38], 3.28 [39], [40], 5.36 [41], 6.55 [42], 6.58 [43], [44], 7.35 [38], [45], 7.36 [46], 7.39 [47] and 7.40 [48]. Also, as the two channels are connected through the orthosteric binding site, we cannot rule out the possibility that ligands could use one route for entry and the other for exit, in the same manner as proposed for the uptake and release of retinal in rhodopsin [23].

Charged residues in ECLs 2 and 3 separate the C1 and C2 channels from each other (Table 1). These residues are D217 and D356 in β_1_AR and D192 and K305, forming a salt bridge, in β_2_AR. Importantly, D217 in β_1_AR and the homologous D192 in β_2_AR are involved in hydrogen bonding interactions with the protonated group of cyanopindolol and carazolol, respectively, during dissociation via both the C1 and C2 channels (see Figures 4 and 5). We hypothesize that these common negatively charged side chains play an important role to attract the ligand to the channels, and to provide the energy to partially desolvate the ligand. Clearly, extracellular ligands must be transferred from the extracellular aqueous environment to the binding site crevice in the TM domain, away from bulk water. Thus, a crucial contribution to the ligand-receptor binding affinity is the desolvation of the ligand. Interestingly, the corresponding residues in β_3_AR are non-bulky hydrophobic amino acids, A197 and G325. These remarkable differences are most likely translated into a different pattern of ligand entry in these receptors.

**Figure 6:**
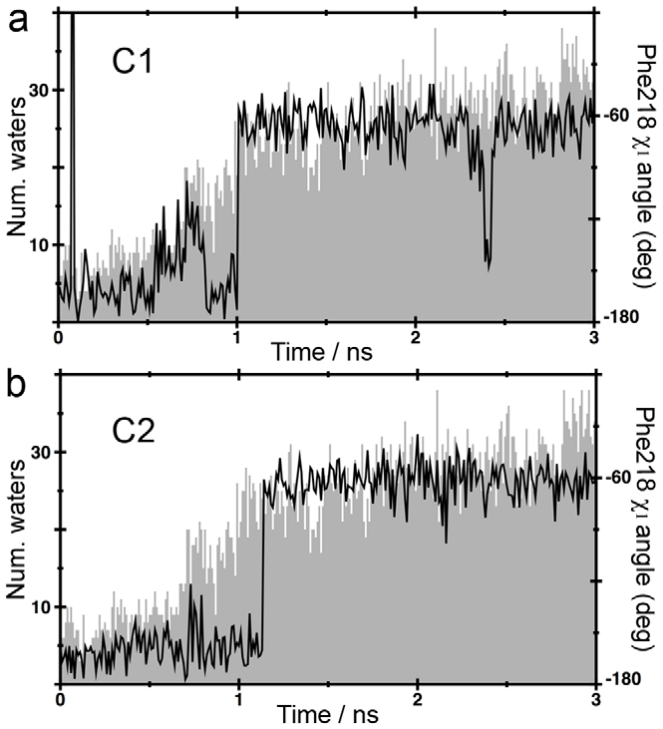
Number of water molecules at a distance of 3 Å from cyanopindolol (grey solid contour) and χ_1_ torsion angle of F218 (black lines) from selected β_1_AR SMD trajectories through channels C1 (panel a) and C2 (panel b).

**Figure 7:**
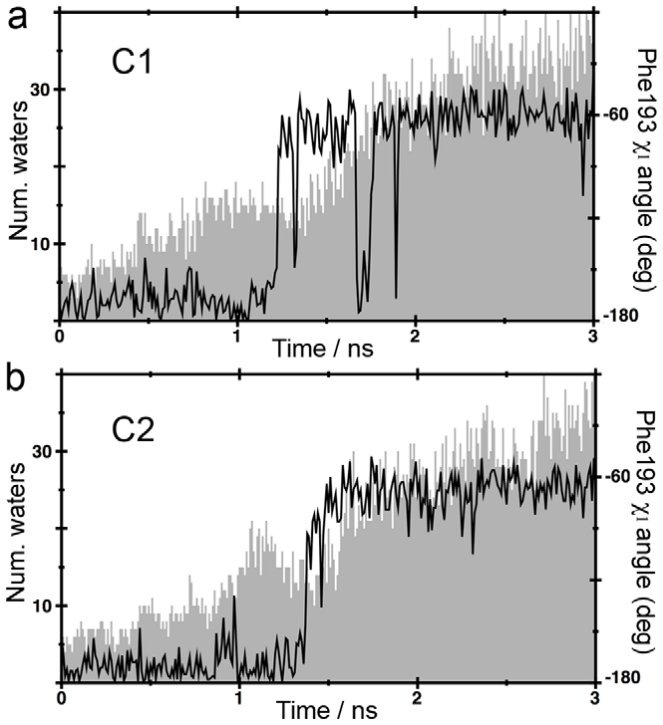
Number of water molecules at a distance of 3 Å from carazolol (grey solid contour) and χ_1_ torsion angle of F193 (black lines) from selected β_2_AR SMD trajectories through channels C1 (panel a) and C2 (panel b).

In addition, the C1 and C2 channels are also delineated by F218 in β_1_AR and F193 in β_2_AR, located in ECL2, and F359^7.35^ in β_1_AR and Y308^7.35^ in β_2_AR, located in TM7 (depicted by solvent surfaces in Figures 4 and 5). Previous MD simulations on β_2_AR have suggested that F193 is able to achieve different conformations [12], [22]. These features were reproduced in our simulations, as we observed a rotation of both the F218 and F193 side chains (black traces in Figures 6a (β_1_AR, ligand exit through C1), 6b (β_1_AR, ligand exit through C2), 7a (β_2_AR, ligand exit through C1) and 7b (β_2_AR, ligand exit through C2) that parallels the transition of the ligands from the TM bundle into the solvent. However, in contrast with previous works, we observed that the conformational changes of F218 and F193 in ECL2 correlate with an increase in the number of water molecules around ligands during dissociation (grey contour in Figures 6a, 6b, 7a and 7b). Based on this observation, we suggest a novel role for these residues: we hypothesize that in the process of ligand entry F218 and F193 serve as a floodgate by removing the water solvent shell around the compounds during binding.

The extraordinary variability in length and amino acid composition of the extracellular loops across the GPCR superfamily generates a wide recognition space for ligands with very diverse chemical scaffolds. For instance ECL 2 of rhodopsin, formed by two β-strands, buries the binding site from the extracellular environment, whereas ECL 2 of CXCR4, also formed by two β-strands, fully exposes the binding site to the extracellular environment. In contrast, a helical segment forms ECL 2 of the β_1_- and β_2_- adrenergic receptors. This α-helix is probably not conserved in the other members of the biogenic amine receptor family, as it was not found in the structure of the dopamine D3 receptor. It was recently shown that ECLs 2 and 3 of the β_2_-adrenergic receptor exist in three distinct conformations depending on the type of ligand bound to the TM core (neutral antagonists, agonists, or inverse agonists) [12], [22]. Thus, this extracellular domain of the receptor plays a key role in receptor activation. We hypothesize that small molecules binding to these secondary-binding pockets, in the extracellular domain, might act as allosteric modulators.

## Methods

### Molecular models and identification of ligand access channels

The high-resolution crystal structures of the β_1_AR [19] and β_2_AR [20] were obtained from the Protein Data Bank (PDB entries 2VT4 and 2RH1 respectively). MODELER [49] was used to transform the starting coordinates of the turkey β_1_AR (UniProtKB: P07700) to the human sequence (UniProtKB: P08588). It is important to note that major differences between turkey and human sequences are present in the N- and C-terminal regions (e.g. human β_1_AR have an N-terminal domain 17 residues longer). The notation of the β_1_AR amino acids in the manuscript corresponds to the human sequence. CAVER [29] was used to determine channels connecting the ligand binding site to the extracellular surface in snapshot structures (every 0.5 ns) along the equilibration period (see below). The initial state for cavities search was at the center of mass of the ligands and a grid spacing of 0.5 Å was used. This approach leads to the identification of two channels in both receptors (C1 and C2 in Figure 1). In addition, we include two inter-helical channels (C3 and C4) calculated by the same procedure for the GPCR opsin [23] and superimposed onto the βARs coordinates. These “rhodopsin-like” channels, however, were not detected by CAVER in the βARs structures.

### Molecular dynamics (MD) simulations

The β_1_AR and β_2_AR human receptors in ligand bound conformation and nine internal water molecules in the P^6.50^/D^2.50^/N^7.49^/Y^7.53^ environment [50] were embedded in a pre-equilibrated lipid bilayer consisting of 282 molecules of 1-palmitoyl-2-oleoyl-*sn*-glycerol-3-phosphatidylcholine (POPC). These crystallographic water molecules did not displace significantly from their starting positions during the simulations (data not shown). Electroneutrality of the system was achieved by adding chloride ions to fulfill a net charge of zero; then, additional sodium and chloride ions were added to a final concentration of 0.1 mol/L. Simulations were carried out using the NAMD version 2.7 MD package [51] using the TIP3 water model and the CHARMM27 all-hydrogen force field [52]. Atomic charges for carazolol and cyanopindolol were calculated with HF/6-31G* and RESP [53], and compared against the corresponding atom types in the CGenFF [54]. In all cases, we only observed small differences in values, while the signs of the charges were always maintained. Long-range electrostatic interactions were calculated using the particle mesh Ewald (PME) method [55]. Initial coordinates were optimized by energy minimization. After geometry optimization, the temperature of the systems was raised in 30.000 steps by temperature reassignment method followed by 10 ns of equilibration at 300 K and constant pressure.

### Steered molecular dynamics (SMD) simulations

The SMD method implemented in NAMD [24] was used to simulate ligands dissociation. The directions of the applied forces (reaction coordinate) were vectors with origin in the center of mass of the ligands and having minimal standard deviation from the path graph nodes defined by CAVER. SMD simulations were performed at constant velocity of 10 Å/ns and the spring constant was set to 250 pN/Å. These parameters were similar to those used previously in biological systems and sufficient to ensure that the work distribution is Gaussian [56]. Each trajectory was carried out until the ligands were displaced towards the receptor surface, and was repeated 6 times. The pulling force *F* at time *t* was calculated according to:
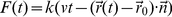
(1)where *k* is the spring constant, *v* is the constant velocity of pulling, *r_0_* and *r(t)* are the ligand center of mass position at initial and current time *t* respectively, 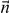 is the direction of the pulling vector. The potential of mean force (PMF) along the reaction coordinate was calculated by the second-order cumulant expansion of the irreversible work measurements [34] according to:
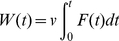
(2)

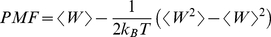
(3)where 〈W〉 is the mean work averaged from the six trajectories, *k_B_* is Boltzmann's constant and *T* is the bulk temperature.

The Jarzynski's equality applied in this study is relative easy to implement compared to other free energy methods such as umbrella sampling. However, it is not exempt of the inaccuracies inherent to the insufficient sampling of the configuration space [57]. Thus, we have only used the PMF profiles as a guideline for the identification of residues involved in interaction with the ligands during the extraction process. Specifically, we do not to aim to compare the theoretical energy values with experimental binding affinities.

## Supporting Information

Figure S1 **Rmsd values of the backbone atoms of β_1_AR (a) and β_2_AR (b) along the trajectories of the MD equilibrium simulations of the receptor-membrane systems.**
(TIF)

Figure S2 **Representative force profiles of ligand extraction along the C1–C4 channels.** Panel a corresponds to the cyanopindolol/β_1_AR complex and panel b corresponds to the carazolol/β_2_AR complex. C1 and C2 correspond to extracellular routes whereas C3 and C4 correspond to routes that lead to the membrane core.
(TIF)
