# Supplementary figures and images for: Molecular Basis of Ligand Dissociation in β-Adrenergic Receptors
Source: PLoS One. 2011 Sep 7;6(9):e23815. doi: 10.1371/journal.pone.0023815 (PMC3168429; doi:10.1371/journal.pone.0023815)

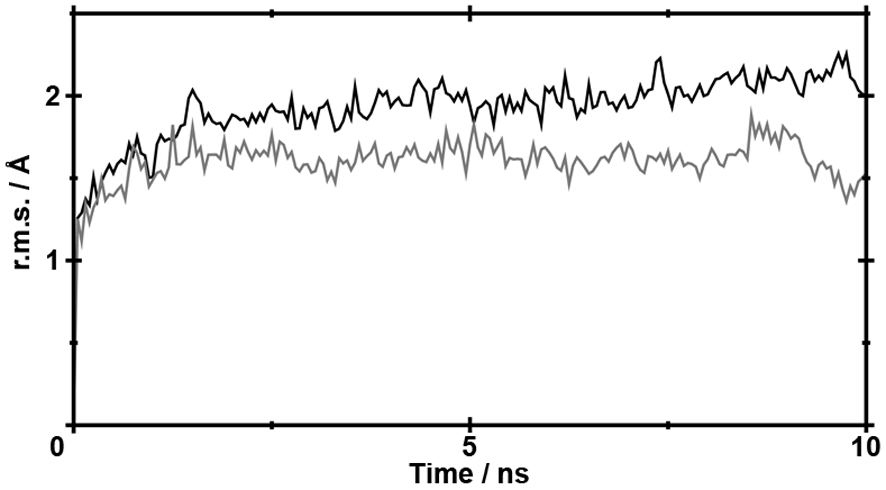

Supplement: Figure S1 — Rmsd values of the backbone atoms of β1AR (a) and β2AR (b) along the trajectories of the MD equilibrium simulations of the receptor-membrane systems. (TIF) [file pone.0023815.s001.tif]

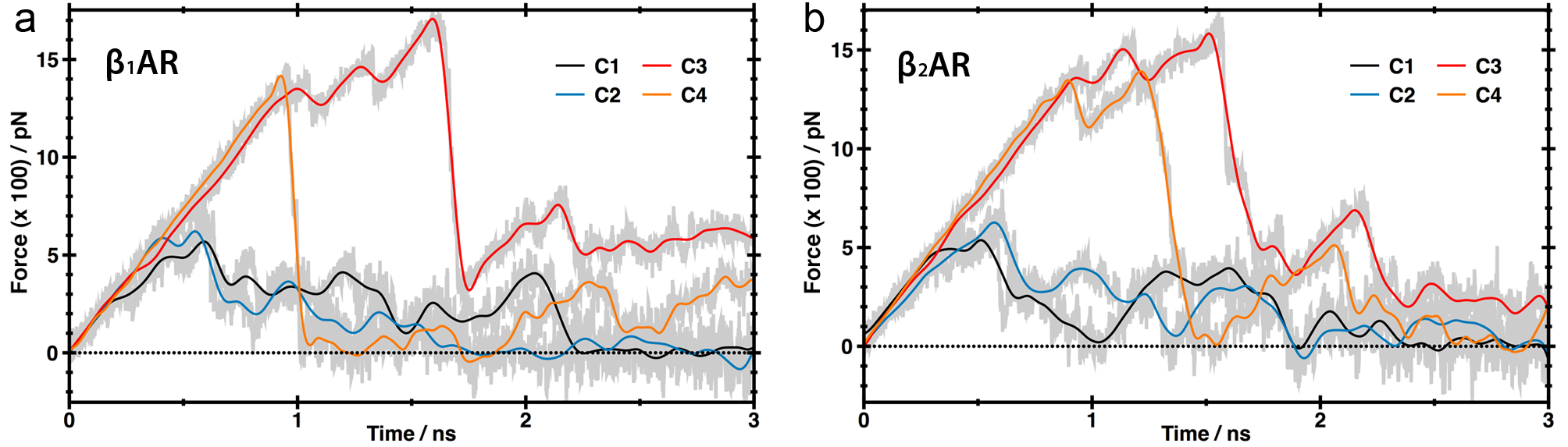

Supplement: Figure S2 — Representative force profiles of ligand extraction along the C1–C4 channels. Panel a corresponds to the cyanopindolol/β1AR complex and panel b corresponds to the carazolol/β2AR complex. C1 and C2 correspond to extracellular routes whereas C3 and C4 correspond to routes that lead to the membrane core. (TIF) [file pone.0023815.s002.tif]
